# Impact of Enhanced Physician's Perception on Optimum Albumin Usage in Adult Critical Care in Qatar

**DOI:** 10.5339/qmj.2023.8

**Published:** 2023-02-21

**Authors:** Safa Al-Rawi, Hassan Mitwally, Edin Karic, Suad Aden

**Affiliations:** Al Wakrah Hospital, Hamad Medical Corporation, Doha, Qatar. E-mail: salrawi@hamad.qa ORCID ID: 0000-0001-5358-2524

Albumin is a colloidal preparation with a limited supply and high cost, which might increase costs if supplied incorrectly. Albumin is derived from human plasma and is available in concentrations of 5, 20, and 25%. It is the first-line option in medical conditions and treatments such as plasmapheresis, paracentesis, extensive burn, spontaneous bacterial peritonitis, and nephrotic syndrome^1^; nonetheless, albumin has been misused for purposes where less expensive options exist. Although colloids do not show a volume-sparing property over crystalloids in these patients due to the capillary leak and other physiological abnormalities, the 5% solution is widely employed to expand and maintain intravascular volume, particularly during fluid resuscitation of severely sick patients. Various large clinical trials revealed contradicting outcomes on the mortality benefit of albumin over crystalloids in critically ill patients.^2–3^ The Surviving Sepsis Campaign recommends that patients with hypotension or a lactate concentration greater than 4 mmol/L should receive a 30 mL/Kg bolus of crystalloid fluid within the first hour with additional albumin fluid resuscitation on an appraisal of subsequent need.^4^

Our department has documented albumin misuse for fluid resuscitation of septic patients who had previously had insufficient or no crystalloid administration; as well as for sedation-induced hypotension or intradialytic hypotension. Our study aims to evaluate optimal albumin utilization six months after implementing an evidence-based guideline and education in selected units of a general hospital in Qatar. We wanted to increase albumin utilization with this quality improvement effort by following the standard evidence-based recommendations. As a result, the institutional review board determined that the project was excluded from scrutiny. The investigation comprised adults older than 14 years who were included in the analysis from two units with high albumin consumption, including one medical and surgical intensive care unit (MICU/SICU). During the pre-implementation phase, baseline data of patients (Group 1) who had been prescribed albumin were assessed by a clinical pharmacist for three months. The data was then examined using evidence-based designed criteria, and the results were provided to the critical care team involved in the prescription of albumin. The team agreed on an action plan including a) educational workshops for albumin's rational use in sepsis and septic shock fluid resuscitation, b) justifying the volume of crystalloids administered prior to prescribing albumin. Physicians were required to write the number of crystalloids provided or if there was another specific reason for prescribing albumin, and c) albumin was removed from the automated dispensing machine override list to avoid unauthorized administration. Post-intervention data were collected for three months to assess the effectiveness of the action plan. The educational sessions and action plan orientation were attended by all ICU physicians.

The following information was extracted from the reports of electronic medical records on dispensed albumin: the amount of prescribed albumin bottles, indications based on guidelines, the volume of crystalloids administered before dispensing albumin, the volume of administered crystalloid, the average cost per patient, and demographic information. The albumin dosage form employed in this audit was 250 ml vials of albumin 5%. Before prescribing albumin to patients with sepsis or septic shock, the criteria for proper use of human albumin are established as a crystalloid dose higher than 25ml/kg. The study excluded patients with liver cirrhosis and resuscitation due to burns.

133 patients treated with 5% albumin were included in this audit, 66 patients before and 58 patients after intervention. The final analysis excluded five patients with 35 albumin vials in the pre-intervention sample, and four patients with 34 vials in the post-intervention sample (burn patients). The majority of patients were men 92 (74.1%), matching the country's overall demographic. In our facility, septic shock fluid resuscitation had the highest albumin use, accounting for 33 (50%) in the pre-intervention period and 33 (56.9%) in the post-intervention phase. This percentage is much greater than the rate reported in a previous albumin-use evaluation conducted on 142 patients (13%).^5^ Table 1a shows the demographic information, including indications for albumin administration and serum albumin in both groups.

As indicated in Table 1b, the number of vials utilized was 319 before the intervention. It significantly decreased by 34.5% to 209 vials after the interventions. Similarly, the number of patients prescribed albumin decreased by 12%, from 66 in the pre-intervention sample to 58 in the post-intervention sample. The total albumin cost was reduced from 18,528 US dollars in the pre-intervention sample to 12,139 US dollars after three months, resulting in a total saving of 6,389 US dollars. The average cost of prescribed albumin for each patient significantly decreased from 280.74 US dollars in the pre-intervention sample to 209.287 US dollars in the post-intervention sample. In the pre-intervention phase, 57.6% of septic shock patients received insufficient or no fluid resuscitation with crystalloid. This pattern continued in the post-intervention phase, with an inappropriate or no fluid resuscitation rate of 60.6%. Studies on albumin use revealed that 50%–70% of prescriptions were inappropriate.^6^ In a Spanish investigation, albumin's irrational use was observed to be more than 90%.^6^ This is consistent with our findings, which achieve a high rate of inadequate prescription of albumin. Misuse of albumin in resuscitation may be due to the mistaken notion that it can correct hypotension faster and with less volume than crystalloids, a claim that has not been substantiated by clinical trials.^2–3^ Noninsurance-based hospitals may be more liberal in their use of albumin since the physician is not often required to justify prescribing such medication despite the availability of a less expensive option. However, we were able to demonstrate a cost savings of about 71.2 US dollars per patient, as well as a 12% reduction in prescriptions. As a result, these findings highlight the significance of a dependable internal protocol for preventing the misuse of albumin and lowering costs. Despite these encouraging findings, inadequate fluid resuscitation in septic shock patients was reported with crystalloid before albumin was similar in the pre/post-intervention groups, 57 and 60.6%, respectively. These findings highlight the importance of enforcing practical clinical recommendations further to promote a more suitable prescription of crystalloid fluid resuscitation.

In conclusion, enhancing physician's perceptions of optimal albumin usage through continuing education, mitigating the volume of crystalloids given before prescribing albumin, and removing albumin from the automated dispensing machine override list resulted in a lower prescription for albumin bottles and a cost saving of 71.2 US dollars per patient, as well as to fewer patients being prescribed albumin.

## Author Contributions

H.M. contributed to the conception, design, and research protocol, in addition to being involved in all aspects of data analysis and manuscript revisions for intellectual content. E.K. contributed to revising it critically for important intellectual content. S.AR. contributed to the first draft of the manuscript. S.A. contributed to the data collection process. All authors approved the manuscript's submission and agreed to be accountable for all aspects of the work. The findings achieved herein are solely the responsibility of the authors.

## Declarations of interest

None declared

## Funding Sources

None declared

## Figures and Tables

**Table 1:** Patient characteristics and albumin-use evaluation

Variables	Before intervention	After intervention
a. Patient characteristics		
Sample, n	66	58
Sex, male, n (%)	47 (71%)	45 (77.6%)
Indications, n (%)		
Sepsis/Septic shock	33 (50%)	33 (56.9%)
Other indications	33 (50%)	25 (43.1%)
Dialysis	4 (6.1%)	6 (10.3%)
Nondialysis	29 (43.9%)	19 (32.8%)
Serum albumin < 20 g/L, n (%)	5 (6%)	7(12%)
b. Albumin-use evaluation		
Albumin bottles prescribed, n	319	209
The average cost of albumin, US dollar/patient	280.5	209.2
Fluid resuscitation status with crystalloids in sepsis/septic shock, %		
No fluid resuscitation	12.12	12.12
Inadequate fluid resuscitation	45.45	48.48
Adequate fluid resuscitation	42.42	39.39
